# Genome Engineering as a Therapeutic Approach in Cancer Therapy: A Comprehensive Review

**DOI:** 10.1002/ggn2.202300201

**Published:** 2024-02-05

**Authors:** Jack Gemayel, Alain Chebly, Hampig Kourie, Colette Hanna, Kayane Mheidly, Melissa Mhanna, Farah Karam, Daniel Ghoussaini, Paula El Najjar, Charbel Khalil

**Affiliations:** ^1^ Faculty of Sciences Balamand University Beirut Lebanon; ^2^ FMPS Holding BIOTECKNO s.a.l. Research and Quality Solutions Naccash Beirut 60 247 Lebanon; ^3^ Center Jacques Loiselet for Medical Genetics and Genomics (CGGM), Faculty of Medicine Saint Joseph University Beirut Lebanon; ^4^ Higher Institute of Public Health Saint Joseph University Beirut Lebanon; ^5^ Faculty of Medicine Saint Joseph University Beirut Lebanon; ^6^ Faculty of Medicine Lebanese American University Medical Center Rizk Hospital Beirut Lebanon; ^7^ Sheikh Shakhbout Medical City Abu Dhabi UAE; ^8^ Faculty of Medicine Paris Saclay University 63 Rue Gabriel Péri Le Kremlin‐Bicêtre 94270 France; ^9^ Faculty of Medicine Balamand University Beirut Lebanon; ^10^ Department of Agricultural and Food Engineering, School of Engineering Holy Spirit University of Kaslik Jounieh 446 Lebanon; ^11^ Reviva Regenerative Medicine Center Bsalim Lebanon; ^12^ Bone Marrow Transplant Unit Burjeel Medical City Abu Dhabi UAE; ^13^ Lebanese American University School of Medicine Beirut Lebanon

**Keywords:** cancer, CAR‐T, genome editing, immunotherapy, therapeutic tool

## Abstract

Cancer is one of the foremost causes of mortality. The human genome remains stable over time. However, human activities and environmental factors have the power to influence the prevalence of certain types of mutations. This goes to the excessive progress of xenobiotics and industrial development that is expanding the territory for cancers to develop. The mechanisms involved in immune responses against cancer are widely studied. Genome editing has changed the genome‐based immunotherapy process in the human body and has opened a new era for cancer treatment. In this review, recent cancer immunotherapies and the use of genome engineering technology are largely focused on.

## Graphical abstract

Genome editing will change the future of cancer treatment, from generalized cancer treatment strategies toward a more personalized approach in cancer treatment, based on patient's genome, immune status, and the genetic profile of the tumor.

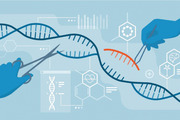

## 1 Introduction

The last decades have seen remarkable and significant advances in cancer research. The prospect for a definite therapy is however still elusive.^[^ ^1^ ^]^ Progress continues to be made, yet the disease remains the second most prevalent cause of death in the world.

Having hundreds of different types, each with diverse genetic alterations, cancer is a remarkably intricate disease. The genetic makeup of cancers varies, not just between the different types, but also within each category and within individual tumors. The basis of cancer is mutations and their accumulations. These mutations either turn on or turn off genes that control tumor development directly or indirectly, promoting unregulated malignant cell proliferation. The complex tumor microenvironment is immunosuppressive and contains a wide variety of cell types with various mutation patterns.^[^ ^1^ ^]^ Moreover, DNA modifications can turn on oncogenes into active proteins and turn off tumor suppressors. Those changes can also alter the epigenome, which regulates genes’ expression and genomic stability.^[^ ^2^ ^]^ On another level, due to persistent screening and diagnostic limitations, many cancer types remain challenging to treat. When cancer is not diagnosed at an early‐stage, metastatic tumors become more likely to present additional mutations, showing different responses to therapy.^[^ ^1^ ^]^ Furthermore, the accumulation of mutations over time might make tumors resistant to treatment. Genetic diversity affects the aggressiveness of tumors and their response to therapies.^[^ ^1^ ^]^

Studies have shown that cancer is a disease of the cell, relying on modifications to the metabolism, cellular structure, and motility to proliferate in unfavorable conditions.^[^ ^3^ ^]^ Almost every aspect of cellular life is governed by genes, producing the needed proteins for cellular growth, function, and division.^[^ ^4^ ^]^ A faulty gene can result in a faulty protein which is unable to carry out its intended function.^[^ ^5^ ^]^ A gene's absence or over‐activity can affect normal biological processes. Gene therapy aims to solve such issues by addressing their root causes. To understand how various forms of cancer develop and what distinguishes cancer cells from healthy tissue, researchers are now exploring the tumor microenvironment, that is found around cancer cells and reportedly encourages its growth and thwart the immune system.^[^ ^6^ ^]^ Adoptive cell therapies for cancer are preferable to conventional therapies, like chemotherapy and radiation, since they are more specifically directed against malignant tissue while sparing healthy cells.^[^ ^7^ ^]^ In all cell‐based immunotherapies, immune cells are expanded ex vivo and then infused back into patients, increasing the body's supply of immune cells that can fight cancer.^[^ ^1^ ^]^ A standardized treatment is hard to develop, making it difficult to create precise study models.^[^ ^1^ ^]^ Cancer research is said to be a dynamic field.

Gene therapy is a technique that alters the genetic code within a person's cells, serving as a method for treating or preventing various conditions, including cancer.^[^ ^3^ ^]^ Gene therapy seeks to manage a tumor's altered gene or a genetic alteration to inhibit malignant proliferation. Gene therapy may be divided into four categories: gene editing, gene replacement, gene addition, and gene inhibition.^[^ ^6^ ^]^

The development of more efficient treatment options and better outcomes for cancer patients depend on our ability to grasp how genomic changes, cellular adaptations, and changes to the microenvironment, influence the initiation, progression, and therapeutic response of specific cancers.^[^ ^2^ ^]^ In this review, we attempt to provide a comprehensive review of the literature regarding genome editing tools and their potential implications in cancer.

## 2 Cancers and Genetics

Cancer is a disordered cell state characterized by uncontrolled divisions and invasion of other tissues. This mainly comes from gene mutations or genomic alterations.^[^ ^8^ ^]^ Recent evidence also suggests that cancer results from a dysregulated metabolism of cells leading to the development of tumors.^[^ ^9^ ^]^ However, the theory of cancer being considered a genetic disorder of a multitude of mutations that run cancer progression still applies.^[^ ^9^ ^]^ Most of these genetic changes are acquired and occur due to microenvironmental exposures or cell aging but can also arise from genetic mutations.^[^ ^10^ ^]^ So, genetic mutations cause inherited cancer syndromes making patients at a greater risk for cancer development. The most common cancer syndromes include Hereditary Breast and Ovarian Cancer syndrome, Lynch syndrome, Li‐Fraumeni, Familial Adenomatous Polyposis, Multiple Endocrine Neoplasia, Retinoblastoma, and Von Hippel‐Lindau.^[^ ^11^ ^]^ We will start by briefly mentioning the first three syndromes cited for a better understanding of the inherited cancer syndromes and their genetic component along with their cancer progression involvement. And in a later stage, we will be discussing the main types of genetic changes in cancer.

### 2.1 Family Cancer Syndromes

Also known as inherited or genetic cancer syndromes, family cancer syndromes represent ≈5 to 10% of all cancers. It's when a mutated gene can pass from a generation to another, making individuals more likely to develop cancer during their lives, generally at a younger age.^[^ ^12^ ^]^ Nevertheless, being born with an inherited genetic defect doesn't necessarily imply that the person will develop cancer; but they have a higher risk of it. This typically defines the genetic predisposition to cancer.^[^ ^13^ ^]^

#### 2.1.1 Hereditary Breast and Ovarian Cancer (HBOC) Syndrome

5 to 10% of all breast and ovarian cancers are attributed to the Hereditary Breast and Ovarian Cancer syndrome (HBOC). This syndrome is defined by the National Cancer Institute as a hereditary disorder where the probability of developing breast or ovarian cancer is higher than normal. A susceptibility and predisposition to these cancers must be considered when several HBOCs are found within the same pedigree, or when it appears at a relatively young age (<50 years of age).^[^ ^14^ ^]^ Interestingly, those cancers are largely found in young women and are seen in men in some cases.^[^ ^15^ ^]^

Germline mutations in *BRCA1* or *BRCA2* genes represent 20% of HBOCs genetic mutations. The risk to develop HBOC in women with *BRCA1* or *BRCA2* is very high. These genes play a major role in maintaining genomic stability. They play a major role in repairing DNA double‐strand breaks via homologous recombination repair. They also control the centrosome dynamics, cytokinesis, and chromosomes’ segregation. *BRCA1* or *BRCA2* stabilize the genome in the cell cycle, temporally and spatially. In addition to that, *BRCA1* is reportedly involved in other functions, such as healthy embryonic development.^[^ ^14^ ^]^ Mutations in both genes can generate truncated encoded proteins.

In addition to *BRCA1* and *BRCA2*, genetic alterations of other genes are associated with HBOC, such as *ATM*, *BRIP1*, *CHEK2*, *RAD50*, *RAD51C*, *TP53*, *PTEN*, *STK11*, *CDH1*, and others. These genes play a role in homologous recombination or genomic stability pathways; leading to HBOC development when mutated.^[^ ^16^ ^]^

#### 2.1.2 Lynch Syndrome (Hereditary Non‐Polyposis Colorectal Cancer)

Lynch syndrome is known to be the most common genetic cancer syndrome that increases a person's risk for colon cancer. Five percent of colorectal cancers are due to inherited cancer syndromes of which 3% are attributed to Lynch syndrome. The prevalence of this syndrome is ≈1/440 in the general population.^[^ ^17^ ^]^

Lynch syndrome is characterized by an autosomal dominant inheritance with a high penetrance, and it appears at an early age. Its genetic mechanism consists of an alteration of the Mismatch Repair (MMR) pathway, which physiologically corrects errors that arise during the DNA replication or recombination.^[^ ^18^ ^]^ This system is also responsible of blocking the recombination between related but non‐identical sequences and acts as a barrier to chromosomal rearrangements.^[^ ^19^ ^]^ The MMR pathway can be affected by a constitutional deleterious mutation in one of the DNA MMR genes. Defective MMR system results in length variation of short tandem nucleotide repeats, also known as microsatellites.^[^ ^19^ ^]^ The microsatellite instability (MSI) is a hallmark frequently detected in Lynch Syndrome. The MMR defect observed is mainly due to mutations in four genes: *MSH2*, *MLH1*, *MSH6*, and *PMS2*. *MSH2* and *MLH1* are most frequently found to be mutated, accounting for ≈90% of the cases. The molecular diagnosis of Lynch Syndrome is confirmed by the presence of constitutional heterozygous pathogenic variants in *MLH1*, *MSH2*, *MSH6*, or *PSM2* genes, or also deletions in the *EPCAM* gene.^[^ ^19^ ^]^

#### 2.1.3 Li‐Fraumeni Syndrome (LFS)

The Li‐Fraumeni Syndrome is a rare inherited syndrome, caused by germline mutations in the *TP53* gene. This results in an increased risk of sarcoma, leukemia, breast, adrenal cortex, and brain cancer. The lifetime risk to develop a cancer in individuals with LFS is above 90% for women and above 70% for men.^[^ ^10^ ^]^ In people with LFS, the risk of cancer is increased in children and young adults, as well as for survivors of a primary cancer.^[^ ^10^ ^]^ When it comes to the LFS diagnosis, three clinical criteria must be present: a diagnosis of sarcoma before 45 years old, a first‐degree relative with a diagnosis of cancer before 45 years old, and a first or second‐degree relative presenting with any cancer diagnosed before 45 years old, or a sarcoma diagnosed at any age.^[^ ^20^ ^]^ The LFS is inherited in an autosomal dominant manner. It is enough to have a heterozygous germline pathogenic variant in *TP53* gene to develop this syndrome.^[^ ^21^ ^]^

So genetic mutations and changes strongly play a role in the development and progression of cancers. Understanding those changes is crucial for the development of therapies that are targeted and personalized. Below is a brief review of the main types of genetic changes seen in cancers.

### 2.2 Types of Genetic Changes in Cancer

There are numerous types of genetic changes affected by cancer. For instance, oncogenes are proto‐oncogenes presenting mutations. Proto‐oncogenes are normal genes who control cell growth. They have the capacity to be switched‐ on and off to control cell growth according to cellular signals.^[^ ^21^ ^]^ However, once mutated, oncogenes are always switched‐on, stimulating cell growth in an uncontrollable way.^[^ ^22^ ^]^ Another type is the tumor suppressor genes. Their role is to slow cell growth and division, repair DNA mistakes and initiate apoptosis or programmed cell death. These genes work properly when they are switched‐on.^[^ ^23^ ^]^ However, they are switched‐off when mutated.^[^ ^23^ ^]^ Therefore, cells continue to grow and evade apoptosis, even in presence of severe DNA damages.^[^ ^24^ ^]^ Moreover, when DNA repair gene are mutated, they lead to chromosomal or molecular aberrations, and genetic instabilities, allowing cancerous development.^[^ ^25^ ^]^

Epigenetic changes play a major role in cancer occurrence. They determine which genes should be switched‐on or off in a very orchestrated manner.^[^ ^26^ ^]^ Taking control of the epigenetic regulation allows cancer to control cell growth and development.^[^ ^27^ ^]^ One epigenetic modification is the DNA methylation mainly on CpG islands.^[^ ^28^ ^]^ Aberrant DNA methylation patterns have been reported in many types of cancer. For instance, a hypermethylation of CpG islands on p53 promoter leads to an inactivation of p53 protein,^[^ ^28^, ^29^ ^]^ and a CpG hypermethylation on the promoter of *hTERT* (human Telomerase Reverse Transcriptase) leads to telomerase activation in cancer.^[^ ^30^ ^]^

In addition, histone modifications, such as acetylation, methylation, phosphorylation, and others, have significant impacts on gene expression and chromatin compaction. Aberrant patterns of histone acetylation, particularly the loss of histone H4 lysine 16 acetylation, are commonly observed in human cancers. Histone phosphorylation, on the other hand, plays a vital role in DNA damage response pathways, and its loss in cancer leads to increased sensitivity to ionizing radiation and genomic instability.^[^ ^30^, ^31^ ^]^

By targeting the genetic mutations and alterations that drive cancer growth, genome engineering technologies have been showing increased potential. Here are some key genome engineering technologies and approaches used in cancer treatment.

## 3 Genome Engineering Technologies for Cancer Treatment

### 3.1 Gene Editing Tools

Gene‐editing technologies have an exceptional potential in treating, curing, or even preventing a variety of diseases, inherited, or acquired, including cancer. The characteristic of these technologies is the flexibility in modifying a genome at a specific sequence. The purpose of that is to either knockdown or restore genes, insert a certain therapeutic sequence, or reverse mutations. Those technologies are wide and their implication in clinical trials is expanding.^[^ ^32^ ^]^ Below we discuss several gene editing tools including CRISPIR‐Cas‐9, Mega nucleases, TALENs, and Zinc finger nucleases.

#### 3.1.1 CRISPR‐Cas‐9

The CRISPR/Cas9 has been remodeled from a system that guards bacteria against plasmid transfer and phage infection, to an RNA‐guided DNA targeting system for genome editing.^[^ ^33^ ^]^ So, it is a promising tool to rectify, delete, or insert the sequence of any mutated or abnormal gene by in vivo or in vitro methods.^[^ ^33^ ^]^ On CRISPR/Cas9 gene‐editing platform, the CRISPR/dCas9 based epigenetic therapy, is an emerging version of precision cancer therapy adapted from the prokaryotic CRISPR‐Cas system. It has been reported to have the full potential to be translated for clinical indications.^[^ ^34^ ^]^ CRISPR/dCas9 is an endonuclease deficient system. It can devoid the side effects related to DNA alteration techniques since it binds to the DNA without cleaving it. It can simultaneously provide an excellent target specificity through well designed single guide RNA (sgRNA) based selective locus‐specific DNA binding property.^[^ ^35^ ^]^

CRISPR‐Cas9 system was investigated in colon cancer epigenetic therapy.^[^ ^36^ ^]^ HIV‐based lentiviral vectors: origin and sequence differences

In this context, SARI (suppressor of activator protein 1, regulated by IFN) that functions as a tumor suppressor and is inactivated in many cancers, including colon cancer.^[^ ^37^ ^]^ The dCas9 system (dCas9‐multiGCN4/scFv‐TET1CD‐sgSARI) targeting the SARI promoter region was constructed with DNA demethylation epi‐effector TET1CD which facilitates the activation of the *SARI* gene.^[^ ^37^ ^]^ This study demonstrated that the created system resulted in the substantial reactivation of *SARI* expression. This significantly reduced the tumor volume and weight of HCT116 cells. Additionally, a phase 1 clinical trial done by Stadtmauer et al. in humans, using a Cas9 system, was done^[^ ^38^ ^]^ in 2 patients with multiple myeloma and one with refractory metastatic sarcoma that had not responded to previous therapy. CRISPR/Cas was employed to remove two genes encoding endogenous TCR, TCRα and TCRβ, and PDCD1 encoding PD‐1 in T‐cells. Plus, the system inserted a synthetic cancer‐specific TCR gene (NY‐ESO‐1) into T cells to identify cancer cells. The edited T‐cells were then transfused back into the patient. Clinical results were promising, and tumor regression was achieved, lasting 4 months, proving feasibility of this novel approach.^[^ ^38^ ^]^ Moreover, CRISPR‐Cas9 could be a potential approach in editing oncogenes in TNBC, or triple negative breast cancer. The system may be used to identify the tumor suppressor genes of TNBC as well as overcoming drug resistance for the TNBC treatment.^[^ ^33^ ^]^

**Figure 1:**
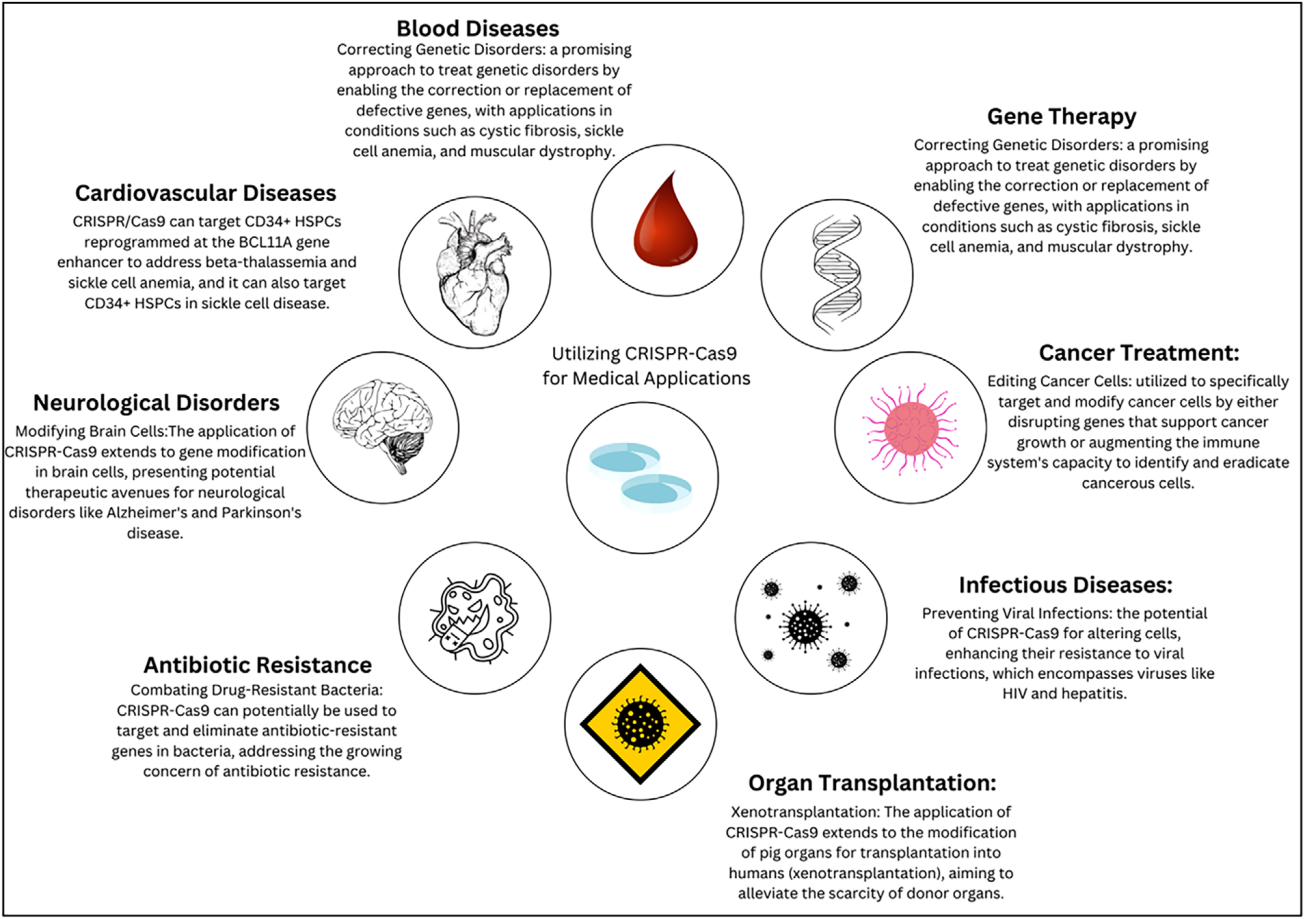
Exploring the Versatility of CRISPR‐Cas9 in Modern Medicine.

Nevertheless, CRISPR/Cas‐9 is not without any limitation or challenge. In fact, the main ones include off‐target effects, non‐specific cleavage at the target, and a lack of potential delivery system. CRISPR/Cas‐9 remains however a potential in gene therapy (**Figure** **1**).

#### 3.1.2 Mega‐Nucleases (MNs)

Mega‐nucleases (endo‐deoxy‐ribonucleases), denoted as MegNs or MNs, can be plainly described as homing endonucleases,^[^ ^39^ ^]^ or big DNA excising machines. Their vast ability to excise big chunks of nucleic acid bases made them a pivotal tool among other molecular techniques.^[^ ^40^ ^]^ The mega‐nucleases through their specific recognition and excision mechanisms allow the specific cleavage of target double‐stranded DNA (ds‐DNA) in a less toxic manner.^[^ ^41^ ^]^ Additionally, MegNs cleave the ds‐DNA through their endonuclease domain and allow the lateral mobility of genetic material between cells in a process known as homing.^[^ ^42^ ^]^ These properties make mega nucleases of a great value in gene therapy. Even though the mega nucleases have a wide implication among various molecular technologies, they did not gain more success in the field of cancer research due to the appearance of new advances in this field.^[^ ^41^ ^]^

#### 3.1.3 Transcription Activator‐Like Effector Nucleases (TALENs)

Transcription activator‐like effector (TALE) nucleases and TALENs are among the first basic genome editing technologies that emerged in 2010 which target DNA genomes with significant specificity.^[^ ^43^ ^]^ TALE is a protein that is synthesized by a plant bacterial pathogen (the Xanthomonas) that is fused to a catalytic domain of the restriction endonuclease FOk1.^[^ ^44^ ^]^ These nucleases trigger a double stranded DNA Break (DSB) at a specific site recognized by the TALE domain. Subsequently, a mutation is induced by the cells own repair systems.^[^ ^45^ ^]^ These include non‐homologous end‐joining (NHEJ) repair, single strand annealing (SSA), and homology directed repair (HDR) if a precise repair template is used. TALE protein consists of 3 domains, with the central repeat domain containing highly variable amino acids at positions 12 and 13, named repeat variable di‐residues (RVDs).^[^ ^46^ ^]^

TALENs could bind precise sites in complex mammalian genomes, but the binding site should always start with a T base, which might be a limiting factor in some cases.^[^ ^47^ ^]^ A Library of 18740 human protein‐coding genes is available for the TALE repeats to be assembled, which further emphasize the ease of access and use of this technology.^[^ ^48^ ^]^

TALENs can be utilized as RNA, DNA, or proteins. They can be delivered through physical direct methods, or through viral and non‐viral vectors.^[^ ^49^ ^]^ TALENs not only can be used to transfer new genetic material, but to alter the expression of existing genes by carrying transcription repressor or activator domains. Furthermore, TALEs can be fused to epigenomic modifiers to alter DNA methylation state and modulate target genes expression.^[^ ^50^ ^]^ TALENs has achieved numerous genome‐editing applications in agriculture and livestock. Their clinical applications remain limited in humans, especially after the shift toward CRISPR‐Cas systems. Nevertheless, it is worth noting that in 2015, TALEN‐edited CAR T‐cells successfully treated 2 patients from leukemia.^[^ ^51^ ^]^

#### 3.1.4 Zinc Finger Nucleases (ZFNs)

Zinc Finger Proteins or ZFNs are engineered hybrid heterodimeric proteins used for site specific genome editing.^[^ ^39^ ^]^ They are made of zinc finger domains, a 3–6 peptides sequence, that bind to specific gene sequences of 3–6 base pairs. When 2 ZFPs fuse with a FokI (*Flavobacterium okeanokoites*) endonuclease I, they induce a DSB at a targeted site.^[^ ^39^ ^]^ Several trials have investigated the use of ZFN in modifying oncogenes or mutant tumor suppressor genes. Zinc Finger Nucleases aim to implement this first‐generation of gene editing in the oncology field. The P53 downregulation, in addition to the tumor angiogenic factors like VEGF‐A, were explored in k562 cells.^[^ ^52^ ^]^

Herman et al. tried to replace a mutant *TP53* with the wild type *TP53* in two cancer cell lines using a liposomal delivery system. The success rate was, however, very low. Another possible use of ZFNs in cancer treatment is enhancing the T‐cell mediated antitumor activity in patients with glioblastomas. It is based on the fact that glucocorticoids can induce T‐cells apoptosis by binding to a receptor on Cytolytic T cells.^[^ ^52^ ^]^ ZFNs were used by Reik et al. to knock down this receptor, thus enhancing immune response to tumor cells in the presence of glucocorticoids.^[^ ^53^ ^]^ In 2018, ZFN were used in a study to target HPV‐16 E6 oncogene; however, the results showed very low editing yield.^[^ ^54^ ^]^ Furthermore, Ding et al. transfected Siha and Hela cell lines with ZFN18‐E7‐S2 to knockdown the expression of E7 oncogene. A significant growth inhibition of treated cells with ZFNs was reported. Another experiment evaluated the tumorigenicity in xenograft injected nude mice. ZFN16‐E7‐S2 and ZFN18‐E7‐S2 were then administrated intratumorally using in vivo transfection reagent leading to smaller tumor growth.^[^ ^55^ ^]^

Another subset of genome engineering technologies for cancer treatment would be the gene delivering technologies.

### 3.2 Gene Delivery Technologies

Aside from gene editing tools, there are some other methods of genome engineering technologies used for cancer treatment and those include the gene delivering technologies. Viral vectors, Bactofection, and Chemical based non‐viral vectors, are potent tools that deliver genes into cancer cells to try and reverse the defect present.

#### 3.2.1 Viral Vectors Used for Gene Delivery

Numerous ongoing clinical trials are evaluating the use of vectors for cancer treatment.^[^ ^44^ ^]^ The most efficient viruses for gene delivery and that are under preclinical and clinical trials include: adenoviruses, adeno‐associated viruses, retroviruses, and lentiviruses.^[^ ^56^ ^]^

Adenoviruses are capable of infecting urinary, gastrointestinal and keratoconjunctival tissues in humans. Replication‐competent adenovirus vectors are reportedly used in oncolytic cancer therapy, while non‐replicative deletion mutants are gene delivery vehicles.^[^ ^57^ ^]^ Adenoviral vectors have broad tropism and do not integrate in human genome but remain episomal. At the end, they fit into scalable production systems.^[^ ^57^ ^]^ Human ad2 and ad5 serotypes are the most used.

Infection initiates with interaction between the cell surface localized coxsackievirus‐Ad receptor (CAR) and the distal domain of the virus capsid fiber. In addition, many other receptors for entry of Ads have been found, such as CD46, DSG2, and sialic acid.^[^ ^58^ ^]^

Due to the widespread of adenovirus seropositive in human population, and the lifelong immunity provided by T cells, these vectors have compromised potencies. Adenoviruses are known to cause cytokine storms and death even with gutless recombinant adenovirus vectors limiting their use in human gene therapy.^[^ ^59^ ^]^ However, this immunogenicity can be employed for triggering anti‐tumor immune response in local cancer therapy.^[^ ^59^ ^]^

Currently, Adenovirus vectors are investigated as genetic vaccine carriers for their ability to deliver foreign epitopes to activate host immune response. For example, human Ad5 is used to deliver SARS‐COv‐2 spike glycoprotein to successfully induce humoral response 14 days post vaccination. In addition, HAd26‐ZEBOV/MVA‐BN‐Filo showed promising results as vaccine carrier against Ebola.^[^ ^60^ ^]^ Other Ad‐based vaccines are under development to target human immunodeficiency virus (HIV). Chimpanzee Adenovirus vectors are also designed and used. For instance, ChAdOx1 in present in two different phase 1 trials expressing NP and M1 antigens for Influenza (NCT01818362 and NCT01623518).^[^ ^61^ ^]^ Furthermore, vaccines for cancer prevention are being investigated in clinical trials. In fact, those vaccines are used for example to express tumor associated antigens, like prostate specific antigens, in prostate cancer. Also, the human papilloma virus E6/E7 antigen for Hpv associated tumors, the carcinoembryonic antigen (CEA) for colorectal and pancreatic cancers, and many more.^[^ ^62^ ^]^ Non‐replicative Adenovirus vectors can have multiple applications in cancer therapy. That is feasible by delivering suicide genes,^[^ ^63^ ^]^ by transferring pro‐drug‐converting enzymes to target tissues,^[^ ^64^ ^]^ and by delivering immune‐regulatory genes to stimulate anti‐tumor response. The intrapleural administration of Ad‐delivered interferon (IFN)‐β or IFN‐α−2b to the lungs have been proven to be safe treatments for malignant pleural mesothelioma.^[^ ^64^ ^]^ Currently, an ongoing phase III trial is investigating the use of Ad‐IFN‐α−2b in combination with celecoxib and gemcitabine (NCT03710876).

Also, tropism modified oncolytic adenovirus vectors are used to target solid tumors receptors, like ColoAd1 (enadenotucirev) against colon cancer cell lines (HT29). This hybrid Ad vector harbors the Ad11p (B group Ad virus) backbone and has a near complete deletion of the E3 region, a smaller deletion in the E4 region, and a chimeric Ad3/Ad11p E2B region. Most recently, two independent phase I trials using ColoAd1 to express FAP‐Tac antibody, together with an immune enhancer module (CXCL9/CXCL10/IFNα) (NCT04053283) and anti‐CD40 antibody (NCT03852511), have been initiated for safety validation.^[^ ^61^ ^]^

Importantly, immunogenicity and toxicity remain a major obstacle for the use of Ad vectors in gene therapy and transfer.^[^ ^65^ ^]^

On the other hand, Adeno‐associated viruses (AAV) are non‐replicative viruses with a broad tissue tropism. They are 25 nm icosahedral viruses with single stranded DNA genomes and lacking many key regulatory genes.^[^ ^66^ ^]^ They have not been found to cause pathology in humans. AAV vectors have low transgene capacity, limiting their use in many clinical applications. AAV bind to specific cell receptors, are released in the cytoplasm by endosomes, and then are trafficked into the nucleus where uncoating occurs for the synthesis of dsDNA. The transferred DNA, once uncoated in the nucleus of host cells, remains as an episomal circle for years.^[^ ^67^ ^]^ In addition, AAV capsids can be modified to target specific cell type receptors and deliver checkpoint inhibitors against programmed cell death protein 1 (PD‐1) and HSV‐TK suicide gene in mice xenograft model, resulting in considerable tumor mass reduction when combined with ganciclovir.^[^ ^68^ ^]^

Luxturna, the first US Food and Drug Administration (FDA), approved an AAV vector for the treatment of multiple retinal diseases. It was used in phase 1 and 2 clinical trials, achieved successful short‐term results in some patients with low side effect profile. However, long‐term efficiency remained uncertain, with failure of treatment due to several factors including possible innate immune response activation.^[^ ^69^ ^]^ Other examples would be Muscular diseases, like the Duchenne muscular dystrophy, and heart diseases. Those were targeted with AAVs with yet very limited benefit.^[^ ^70^ ^]^ Nowadays, further trials for the treatment of hemophilia and central nervous system diseases are still ongoing with AAV5 and AAV6 serotypes.^[^ ^70^ ^]^

Currently, AAV vectors are the most successful in treatment of monogenic diseases like spinal muscular atrophy;^[^ ^56^ ^]^ although their use in cancer therapy remains under investigation.^[^ ^71^ ^]^

Lentiviruses (LVs) constitute a genus of the retro‐viridae family and are divided into simple or complex based on their genome organization.^[^ ^71^ ^]^ LVs, mainly HIV‐1 that was extensively studied in the last decade, make a great tool for gene transfer and clinical applications, particularly in ex‐vivo models. LVs can integrate in non‐dividing cells, have acceptable cargo capacity (<9 kbp), reduced the likelihood of being silenced, and have a wide selection of envelope proteins pseudo typing.^[^ ^72^ ^]^ New generation HIV‐1 vectors are devoid of their disease‐causing genes. They are pseudo typed with vesicular stomatitis G glycoprotein instead of their native envelope protein.^[^ ^73^ ^]^ This confers on them an increased productivity, stability, and infectivity, as well as a larger tropism. The delivered transgene RNA sequence is flanked by modified long terminal repeats (LTR), a promoter, a packaging system, and reverse‐transcription elements. This expression cassette is integrated into genome of transduced cells, ensuring long‐term expression in dividing and non‐dividing cells.^[^ ^74^ ^]^ A major complication of integration was the induced genotoxicity and mutagenesis, mainly with early generations lentivirus, which triggered leukemias and other malignancies in human and animal trials.^[^ ^75^ ^]^ Non‐integrating lentiviral vectors were developed to increase the safety and reduce oncogenic potential of LV vectors.^[^ ^76^ ^]^ Clinical applications focus on modifying T and NK cells for the treatment of non‐Hodgkin lymphoma (NHL) and acute lymphoblastic leukemia (ALL). Another lentiviral cancer vaccine, the LV305, is dendritic cell specific and is used to promote immune presentation of New York Esophageal squamous cell carcinoma‐1 (NY‐ESO‐1) cancer antigen.^[^ ^77^ ^]^ This non integrating vector is pseudo typed with Sindbis virus envelope glycoprotein that binds CD209 receptor on DC. In human phase 1 study LV305 vaccination induced CD4+ and CD8+ T‐cells responses against NY‐ESO‐1‐expressing tumors.^[^ ^78^ ^]^

Gamma retrovirus vectorsare derived from murine leukemia virus (MLV) and unlike lentiviruses, they only infect actively dividing cells. The fact that cells are dividing increases the potential risk of insertional oncogenesis.^[^ ^79^ ^]^ Nevertheless, therapeutic use against diffuse large B cell lymphoma was done with axicabtagene ciloleucel (Yescarta, Gilead).^[^ ^80^ ^]^ This vector transfers chimeric receptors to CD19 T‐cells. Another gammaretroviral therapeutic is the vocimagene amiretrorepvec (Toca 511). It encodes yeast cytosine deaminase that converts prodrug 5‐fluorocytosine to toxic 5‐FU in glioma cells.^[^ ^81^ ^]^

#### 3.2.2 Bacterial Mediated Gene Transfer (Bactofection)

Bactofection is the utilization of bacteria for the purpose of introducing genetic material into target cells. It's an approach that has been used in numerous diseases including cancer, leishmaniasis, cystic fibrosis, and others. During this process, bacterial lysis introduces into mammalian cells a plasmid, which is then expressed into a specific target protein.^[^ ^82^ ^]^ More than a century ago, a significant role of streptococci and their toxins in curing inoperable cancers was discovered by William B. Coley, highlighting the role of bacteria in targeting cancer cells.^[^ ^83^ ^]^ A myriad of microorganisms has been considered for bactofection, including Listeria monocytogenes, Escherichia coli, and Salmonella species. Clostridia was among the initial bacteria implemented in cancer therapy; however, due to the high toxigenicity associated with clostridia, the attention was switched toward gram‐negative microorganisms like Salmonella.^[^ ^84^ ^]^

Nontyphoidal Salmonella is an etiological agent of mild cases of gastrointestinal inflammation. For many years, it has been solely regarded as an infectious microorganism with the focus on its harmful role until its anti‐cancerous role came into light.^[^ ^85^ ^]^ The efficiency of Salmonella species (especially S. typhimurium) in fulfilling this role is astounding.^[^ ^86^ ^]^ The antitumor role of S. typhimurium is thought to be in part related to the release of the pro‐inflammatory pathogen‐associated molecular patterns (PAMP). The cytokines that are released in multitude into the vicinity of the tumor leads to the regression of the tumor cells.^[^ ^87^ ^]^ Jazeela et al. stated that the tumor cells provided an anoxic environment, which favored the growth and the multiplication of S. typhimurium due to its facultative anaerobic features. The ability of S. typhimurium to concentrate around tumor cells and invade their environment, made them a great candidate to be used as vectors for targeted cancer gene therapy.^[^ ^88^ ^]^ Salmonella equipped with shRNA targeted against indoleamine 2,3‐dioxygenase 1 (IDO), which is essential for the growth of tumor cells, resulted in the tumor regression.^[^ ^89^ ^]^ The suppression of IDO by engineered Salmonella in polymorphonuclear cells allows for the synthesis of serine proteases, Fas death ligand expression, and the release of reactive oxygen species (ROS). This series of events eventually manifests in massive apoptosis in the tumor cells population.^[^ ^90^ ^]^

Colorectal cancer (CRC), which is the third most common cancer in the world, is shown to be influenced by a range of proinflammatory cytokines.^[^ ^91^ ^]^ IL‐6 for instance promotes invasiveness and metastasis of tumor cells while IL‐8 takes part in enhancing tumors’ angiogenesis.^[^ ^92^ ^]^ Hence, the delivery of cytokines binders through the bacterial vector Lactococcus lactis to the site of the tumor cells can help reduce the potentiating effects of these cytokines on the tumor cells, and eventually the regression of the tumor.^[^ ^93^ ^]^ The plasmid‐transformed and modified L. lactis have dual functions: the first is the recognition of the tumor antigens‐8, and the second is the expression of the cytokines‐binding proteins that render the target IL‐6 and IL‐8 inactive. The production of these two cytokines‐binding proteins is achieved through the introduced dual promoter plasmid (pNZDual), that is delivered and expressed by the bacteria L. Lactis.^[^ ^93^ ^]^ Engineered L. lactis bacteria have displayed strong and specific attachment to the HEK293 cells, which overexpress EpCam and HER2 antigens, targeting additionally the tumor antigens.^[^ ^93^ ^]^

Furthermore, *Escherichia coli* (*E. coli*) presents to be a prominent tool as a bactofection vector. It is both efficient and specific in targeting tumor cells.^[^ ^94^ ^]^ Evidence has shown that the Symbioflor‐2 probiotic *E. coli* were cleared fast in the liver and the spleen but survived only within the tumor itself. This displays a high specificity of the bacteria in targeting the tumors. An additional factor to the selectivity of *E. coli* to the tumor cells, is the expression of synthetic adhesins (SA), that can selectively bind to the tumor cells.^[^ ^95^ ^]^ Plus, in the *E. coli* bacteria, a glucose sensor was engineered by conjoining a chemoreceptor (Trg) with an osmosensor (EnvZ), allowing the bacteria to determine glucose levels, and the metabolic level, in the tumor cells. This in turn enhances the virulence of the *E. coli* against the tumor cells conferring the therapeutic role of bactofection through E. coli against cancers.^[^ ^96^ ^]^ Another highlight of the molecular engineering features in *E. coli* and S. typhimurium, is the production of weakened S. typhimurium (VNP20009) with the expression of *E. coli* cytosine deaminase (CD). It has actually shown a great role in activating the prodrug of 5‐Fluorouracil (5‐FU).^[^ ^97^ ^]^ The enzyme cytosine deaminase converts 5‐Fluorocytosine (5‐FC) into 5‐Fluorouracil (5‐FU), which is an active toxic chemotherapeutic agent. Therefore, the administration of S. typhimurium (VNP20009) strain with 5‐FC results in the production of 5‐FU, serving as a factory to produce 5‐FU from its prodrug 5‐FC.^[^ ^98^ ^]^

Bactofection showed a high efficiency in infecting target cells, especially tumor cells, serving their main role of gene delivery and therapy.^[^ ^99^ ^]^ Bactofection is especially efficient against tumor cells due to chemotaxis and due to the specific surface adhesions between the bacteria and the surface of tumor cells, resulting in a high transfection efficiency.^[^ ^97^ ^]^ However, there is a safety concern that comes with the use of bacteria as vectors for gene therapy. These bacteria can still cause their respective infectious diseases. Plus, and due to the molecular engineering that they undergo, the bacteria can have their virulence enhanced equally against pathologic cancerous cells and healthy cells of the body.

#### 3.2.3 Chemical‐Based Non‐Viral Vectors

Non‐viral vectors have emerged in parallel to viral and bacterial vectors, for their lower immunogenicity, broad spectrum of modification and tropism, and increased availability at lower costs.^[^ ^100^ ^]^ These chemical vectors can be lipid based, peptide based, or polymer based nanocarriers. Furthermore, inorganic nanocarriers like gold particles have been explored.^[^ ^101^ ^]^

In gene delivery, lipid‐based nanocarriers, especially lipid nanoparticles (LNPs) are the most widely explored alternative to viral vectors.^[^ ^102^ ^]^

Liposomes lipid‐based carriers are successfully used clinically in antitumor drugs like Doxil, DaunoXome, Myocet, DepoCyt, Marqibo, and Onivyde.^[^ ^103^ ^]^ Also, LNPs have been FDA approved in many clinical applications like covid‐19 vaccines.^[^ ^104^ ^]^ These closed vesicles have a phospholipid bilayer structure with amphiphilic lipids and a helper lipid like cholesterol, in addition to other modifying agents like polyethylene glycol (PEG). Cholesterol have shown to improve nanoparticle stability and intracellular trafficking, while PEGylation is a well‐known strategy for immune evasion.^[^ ^105^ ^]^ Liposomes can be prepared with thin film hydration method, reverse phase evaporation, solvent (Ethanol/ether) injection, and detergent dialysis. They can be neutral, cationic, or anionic lipids.^[^ ^106^ ^]^ Cationic liposomes (CLs) are easily used as vectors for their electrostatic interactions with genes, and they provide efficient in vitro transfection, high loading capacity, structural flexibility, and easy large‐scale production.^[^ ^107^ ^]^ However, their cationic charge increases their binding and interaction with serum enzymes and increases cellular toxicity.^[^ ^108^ ^]^ Ionizable lipids where cationic heads are replaced with ionizable moiety like 1,2‐dioleoyl‐3‐dimethylammonium propane (DODAP) can bypass this issue. Indeed, at physiologic pH they are neutral, hence less toxic, and in the cytoplasm they are positively charged following endosomal acidification.^[^ ^109^ ^]^

Polymer based nanocarriers are also used as particles and gene delivery vectors. These polymers can be made of chitosan, Hyaluronic acid, polyethylenimine (PEI), polyamidoamine (PAMAM) dendrimers, or poly B‐amino esters (PBAE).^[^ ^110^ ^]^ The positively charged polymer is combined to nucleic acids by electrostatic action. A PEI based vector was used by Wang et al. to deliver therapeutic gene (PRR/sTRAIL) that encodes human tumor necrosis factor related apoptosis inducing ligand for breast cancer treatment.^[^ ^111^ ^]^ In another study, highly branched Poly beta‐amino ester (PBAE) vectors exhibited remarkably higher DNA/siRNA transfection.^[^ ^112^ ^]^ PBAE based vectors were used by Karlsson et al. to effectively deliver SiRNA in vivo and knock down orthotopic tumors derived from human GBM1A implanted in mice. PBAEs demonstrated low toxicity and high safety profile.^[^ ^113^ ^]^ Because vectors made of one biochemical component could not achieve, all the criteria needed for successful transfer of cargo, adequate capacity, long term stability and acceptable safety profile, novel hybrid nanocarriers (HNCs) emerged.^[^ ^114^ ^]^ HNCs are still underdevelopment and further research is needed prior to preclinical and clinical applications.^[^ ^115^ ^]^

Other potential delivery vectors include carbon nanotubes, calcium phosphate nanoparticles, silica, and magnetic nanoparticles and more progress is needed to accomplish optimal gene therapy.^[^ ^116^ ^]^

All in all, non‐viral vectors are simple in theory but remain complex in practice. Challenges on several levels need to be overcome to increase the effectiveness of this gene transfer. Those challenges mainly turn around the production, formulation, and storage of the non‐viral vectors.^[^ ^100^ ^]^

### 3.3 Gene Therapy for Cancer Treatment

The US FDA defines gene therapy as “a technique to modify the host genes, or manipulate the expression of a gene, or change the host cell biological properties for therapeutic use”. Gene therapy is a vast world. From replacing non‐functional genes with normal ones, to silencing mutated genes, and enhancing the expression of healthy ones, gene therapy has come a long way.^[^ ^117^ ^]^ Herein we review some of the gene therapy tools including Oncolytic Virotherapy, Imlygic, Gendicine, Oncorine, and Rexin‐G.

#### 3.3.1 Oncolytic Virotherapy

Oncolytic virotherapy (OV) is a tool that uses viruses to destroy cancer cells by selectively infecting, replicating in, and killing those cells, while keeping normal cells unaffected.^[^ ^118^ ^]^ In fact, early work started on murine tumors where some virus strains have grown in cancerous cells making the tumor less malignant.^[^ ^119^ ^]^ In the first human trials, alive viruses were used, and they proved antineoplastic activity, however with lethal toxicities.^[^ ^120^ ^]^ With the advances in human cell culture and molecular biology, genetic material was more accessible and easier to alter in vitro. That was the beginning of genetic therapy era, by altering existing genes or introducing new genetic material.^[^ ^121^ ^]^ In parallel, the work on vaccines has started, viruses were genetically modified to keep their selective infectious capabilities, deactivate some genes and keeping other ones, to finally get a virus with attenuated pathogenicity while keeping its antineoplastic effect, entering clinical trials by the 1990s.^[^ ^122^ ^]^ Oncolytic viruses therefore offer a dual therapeutic role combining tumor‐specific cell lysis along with immune stimulation, thus acting as a potential in situ tumor vaccine.^[^ ^123^ ^]^

Viruses have specific cellular tropism that favors the target tissues to be infected. For example, HIV damages helper T lymphocytes, Rabies virus targets neurons, and hepatitis B virus infects hepatocytes. Similarly, some viruses can target exclusively some cancer cells.^[^ ^124^ ^]^ Oncolytic viruses can infect tumor cells leaving behind normal healthy cells because of certain signaling pathways, tumor‐specific promoters, or mRNAs.^[^ ^120^ ^]^ For example, to eliminate a virus in normal cells, antiviral signaling pathways such as the IFN pathway are activated.^[^ ^125^ ^]^ This is not the case in cancer cells where those pathways are altered.^[^ ^126^ ^]^ Similarly, having a defective IFN pathway in cancerous cells makes it possible for the Vesicular stomatitis virus (VSV) to replicate, while having the M protein as a virulence factor. Moreover, the Reovirus have a preference toward tumor cells with an overactive RAS signaling pathway, which is usually silent in normal cells.^[^ ^127^ ^]^ Furthermore, viral replication depends on the inhibition of the p53, a key feature of tumor cells.^[^ ^128^ ^]^ So, in a nutshell, oncolytic viruses have a higher specificity for tumor cells in comparison with normal cells. This specificity can be further enhanced by multiple ways like genetically altering the viral capsid protein increasing the binding specificity to tumor cells.^[^ ^129^ ^]^ For instance, tumor cells are known to express the uPAR receptor on their surface, therefore the construct MV‐h‐uPAR was manufactured. It contains an added portion that binds uPAR, to exclusively infect tumor cells with a higher specificity.^[^ ^130^ ^]^ Another way is to insert lysine residues into ciliated proteins to allow viruses to better target tumor cells that have heparin sulfate receptors.^[^ ^131^ ^]^

Although oncolytic virotherapy is a potent tool in cancer therapy, one type of oncolytic virus is certainly not sufficient because of the diversity of cancer cells and cancer tissues. OV should carefully consider which virus to implement to which type of cancer and to which patient. Many viruses are being studied as potential OVs such as measles virus, vaccinia virus, adenovirus, herpes simplex virus, reovirus, and coxsackievirus.^[^ ^123^ ^]^

Adenovirus is used with three modifications to obtain cancer selectivity. Adv can selectively multiply in p53‐muated and in retinoblastoma (pRb) tumor cells if we delete the E1A and E1B 55K genes of the virus. Also, if we partially delete the E3 region, the AdV will be able to encode immunostimulatory transgenes, thus enhancing antitumor immunity. And to improve the infectivity of the virus, we can insert the Arg‐Gly‐Asp (RGD) motif into the HI loop of the AdV fiber protein.

However, the only one that has been approved by the (FDA) and the European commission for oncolytic virotherapy is the genetically engineered herpes simplex virus‐ Talimogene Laherparepvec (T‐VEC).^[^ ^132^ ^]^ The T‐VECis genetically manufactured by deleting ICP34.5 and ICP47 and inserting GM‐CSF.^[^ ^133^ ^]^ The ICP34.5 encodes the neurovirulence factor meaning that its deletion will stop the replication of the virus in neurons and increase it in tumor cells. Placing two copies of GM‐CSF promotes dendritic cell maturation. Besides deleting ICP47 enhances the immune response against tumor cells, since ICP47 encodes an inhibitor of antigen presentation that blocks MHC class 1 antigen presentation to CD8+ T cells.^[^ ^134^ ^]^ Moreover, the vaccinia virus W has proven a natural selectivity to tumors, and the deletion of the thymidine kinase TK showed further specificity to cancerous cells. Furthermore, and as previously mentioned the Reoviruses target tumor cells that overexpress Ras which is key to the replication of the virus. Another example is the Measles virus interacts with host cells through three receptors including the SLAM/CD150. This same receptor is also overexpressed on many hematological malignancies making MeV naturally selective for infecting tumor cells.^[^ ^135^ ^]^

Oncolytic virotherapy uses cancer‐killing viruses as a tool in cancer therapy. It has potential in metastatic cancers. Nevertheless, some areas still need improvement, with a focus on increasing its efficacy, safety, and the optimization of OV delivery.^[^ ^136^ ^]^

#### 3.3.2 Imlygic ‐ Talimogene Laherparepvec

Talimogene Laherparepvec (T‐VEC) is an engineered oncolytic herpes simplex virus type 1 (HSV‐1) with multiple variations conferring it an oncolytic therapy, selectively recognizing, infecting, and destroying malignant cells.^[^ ^137^ ^]^ As previously described, the neurovirulence factor ICP34.5 is inactivated, preventing neuronal effects, which enhances viral multiplication, and is replaced by the GM‐CSF coding sequence. The latter enhances the immune response to the tumor cells by local GM‐CSF production which increases the influx and activation of dendritic cells required for the initiation of a systemic antitumor response.^[^ ^138^, ^139^ ^]^ Moreover, the ICP47 gene is also deleted promoting host immune response and viral immunogenicity. T‐VEC is also altered in rearranging the herpes unique short 11 (US11) gene under an early, rather than the native late, promoter avoiding the rapid viral clearance and thus increasing its life cycle.^[^ ^140^ ^]^ The intratumorally injection of this virus in tumor cells promotes a direct killing of the cells and acts as a source of antigens which locally engages immune cells into the tumor environment.^[^ ^141^ ^]^

The very first clinical trial of T‐VEC was performed in 2006 where a phase 1 study was conducted on 30 individuals with variant types of cancers.^[^ ^142^ ^]^ A safety profile was set, and evidence showed necrosis and inflammation, with the virus located in tumor cells only, and an increase in GM‐CSF. A phase 2 trial was then performed in patients with superficial accessible melanoma with an objective response rate of 26% success.^[^ ^142^ ^]^ Afterward, an OPTIM study was performed in 436 patients with unresected stage IIIB to IV melanomas comparing it to subcutaneous GM‐CSF treatment. In the last report of this study, T‐VEC showed higher DRR (durable response rate) and ORR (objective response rate) than GM‐CSF with an acceptable toxicity profile. Consequently, T‐VEC was officially approved on October 27^th^, 2015, by the FDA.

Numerous new studies are conducted using T‐VEC. It has been found that T‐VEC is unlikely to be transmitted from treated patients to their close contacts.^[^ ^143^ ^]^ Detectable T‐VEC DNA is found at the highest levels right after administration of the treatment, but it declines rapidly after subsequent doses due to humoral responses. Moreover, detectable DNA is mainly present in samples from the surface of injected lesions.^[^ ^143^ ^]^ Real‐life evidence has proved good tumor responses in older patients with a relatively short duration of treatment and feasible costs.^[^ ^144^ ^]^ Also, adding T‐VEC to systemic chemotherapy increases responses in high‐risk early stage TNBC (triple negative breast cancer).^[^ ^144^ ^]^

#### 3.3.3 Gendicine ‐ Recombinant Human P53 Adenovirus ‐ Ad5RSV‐P53

The p53 gene, named guardian of cells, is associated with numerous functions of cells including cell cycle regulation, cell‐fate determination, DNA replication, modification and repair, angiogenesis, anti‐infection, and immunity.^[^ ^145^ ^]^ In human cancers, the P53 gene is widely mutated, over 50% of all cancers have a mutated P53, which promotes tumor proliferation, increasesmalignancy, decreases survival, and induces resistance to chemotherapy. Thus, restoring the wild type p53 in tumors can return the cell cycle arrest and apoptosis mechanisms and diminish chemotherapy resistance.^[^ ^146^ ^]^ Recombinant human adenovirus p53 (rAd‐p53), also known as Gendicine, is a replication‐incompetent recombinant human serotype 5 adenovirus. Replication incompetent means that this vector is capable of inducing host immune responses without replicating inside the host cells. In this recombinant, the virulence region E1 is substituted with the p53 gene.^[^ ^147^ ^]^ Gendicine is the very first gene therapy‐based that is approved by the State Food and Drug and Administration of the People's Republic of China (SFDA) in 2003.^[^ ^148^ ^]^ Briefly, once tumor cells are infected, the virus does not replicate, instead it will provide the p53 gene to the cellsin order to induce cell death, suppress angiogenesis and induce necrosis.^[^ ^146^ ^]^ Gendicine has been applied in several cancers, such as oral cancer and oral leukoplakia with beneficial outcomes and limited side effects.^[^ ^149^ ^]^ Combining it with chemotherapy is also of beneficial for stage III oral cancer patients. However, no international studies were done, and the little studies have done for oral cancers are restricted to the Chinese population, limiting its potential validation.^[^ ^150^ ^]^ Also, in liver cancer, rAd‐p53 is effective, fairly safe, and is usually combined with other therapies.^[^ ^151^ ^]^ In fact, curcumin induces cell cycle arrest and apoptosis of cancer cells by targeting p53 and subsequent MAPK signaling.^[^ ^152^ ^]^ So, the combination of the two therapies synergistically promotes liver cancer apoptosis, by blocking the G2/M phase cycle and suprerssing EMT. In other studies, rAd‐p53 was combined with other therapies comprising 5‐FU administration, transarterial chemoembolization, or N‐Myc proto‐oncogene.^[^ ^153^ ^]^ Other studies have been conducted in f hypopharyngeal squamous cell carcinoma (HPSCC). Patients have been treated with the combination of rAd‐p53 with CRT (chemotherapy) and previous Surgery (complete tumor surgical resection) with improved therapeutic effects. Moreover, it was shown that rAd‐p53 is tolerated by patients and does not increase the side effects of surgery or CRT.^[^ ^138^ ^]^ Nevertheless, few toxicities have been presented like fever, gastrointestinal reactions, and some muscle aches; however, those were considered not severe, yet negligible. Nevertheless, few studies on rAd‐p53 in HPSCC are available, and there is a need for more studies to validate those findings.^[^ ^138^ ^]^ Furthermore, comparable results are present in a study conducted in cases of nasopharyngeal carcinoma (NPC), stressing the importance of combining rAd‐p53 to CRT/RT (radiotherapy). rAd‐p53 enhances the sensitivity of RT in vivo and in vitro.^[^ ^138^ ^]^ Similarly, in a study on pancreatic cancer, the rAd‐p53 was useful to sensitize the SW1990 cells to radiation, increasing the radiosensitivity to those cells by affecting the expression of P21 and Bax.^[^ ^139^ ^]^ P21 is controlled by p53 and plays a key role in growth arrest when cells are subject to radiation. On the other hand, Bax is involved in p53‐mediated apoptosis and is upregulated by p53.^[^ ^154^ ^]^ On another hand, in cervical cancer, rAd‐p53 was also reported to be an efficient therapy.^[^ ^154^ ^]^ As for other cancers, it can also be combined with CT/RT/CRT for better and safer outcomes of this highly malignant cancers (**Table** **1**).^[^ ^155^ ^]^

**Table 1:** Summarizes the pros and cons of different biological vectors.^[^ ^156^ ^]^

Empty Cell	AAV vector (adeno‐associated virus)	AdV vector (adenovirus)	LV vector (lentivirus)	VLP (virus‐like particle)
Cargo size limit	< 4.7 kb	8–37 kb	8 kb	> 8 kb?
Duration of expression	Several years in non‐dividing cells	< 2 months	Several years	< 1 day
Immunological response	‐ Neutralization by antibodies.	‐ Neutralization by antibodies ‐ Cytotoxicity.	‐ Stimulation of innate immunity. ‐ Antibody production against protein components.	‐ Antibody production against protein components.
Pros	‐ High transduction rate. ‐ Derived from a non‐pathogenic virus. ‐ A broad range of target cells depends on serotypes. ‐ Extensive experiences in gene therapy. ‐ A small diameter (20 nm) allows better penetration in tissues.	‐ High transduction rate ‐ Large (8–37 kb) cargo capacity ‐ Minimal genomic integration ‐ A broad range of target cells	‐ High transduction rate ‐ Large cargo capacity ‐ Less immunogenic. ‐ A broad range of target cells depends on the envelope.	‐ Transient expression ‐ Simplistic structure and easy to engineer. ‐ No risk of genomic integration ‐ Superior biosafety. ‐ A broad range of target cells depends on the envelope.
Cons	‐ Pre‐existing antibodies preclude the use. ‐ Low or almost no transduction efficiency from 2nd administration due to neutralizing antibody. ‐ Liver toxicity at high dose (>10^14^ v.g./kg). ‐ Integration of the vector genomic sequence. ‐ Prolonged expression (several months to years) leads to increased off‐target probability.	‐ Pre‐existing antibodies against Adv. ‐ Low transduction efficiency from 2nd administration due to neutralizing antibody. ‐ Cellular damage.	‐ Random integration of cargo genome sequence. ‐ Prolonged expression leads to increased off‐target probability. ‐ Low transduction efficiency from 2nd treatment due to immunogenicity	‐ Possible immunogenicity. ‐ No extensive experience in clinical trials (except for vaccine use).

#### 3.3.4 Oncorine (rAd5‐H101)

Oncorine is the first recombinant oncolytic virus (OV) to have regulatory approval worldwide and the very first OV for clinical use in China. In 2005, it was approved by the FDA for patients with head and neck cancer receiving chemotherapy, and for the treatment of nasopharyngeal cancers. Oncorine is an attenuated serotype 5 adenoviral vector with specific deletions in the E1B‐55K and four deletions in viral E3.^[^ ^157^ ^]^ In general, the E1B protein, product of the E1B‐55K gene, inhibits apoptosis by binding and degrading p53 transcription factors. Therefore, inactivating this gene will inhibit viral proliferation in healthy cells with normal p53 gene function, while being able to replicate in cancer cells with defective p53 function.^[^ ^158^ ^]^

#### 3.3.5 Rexin‐G (Mx‐dnG1)

Rexin‐G, Mx‐dnG1, also named DeltaRex‐G, is a replication incompetent retroviral vector, encoding a dominant negative inhibitor construct CCNG1 gene (cytocidal cyclin G1).^[^ ^159^ ^]^ CCNG1 takes part of the Cyclin G protein family that regulates cell growth. Its specific function is yet to be discovered, however its association with an abnormal expression in cancer cells has been already proven.^[^ ^160^ ^]^ Here comes in place the role of Rexin‐G which inhibits CCG1 in tumor cells leading to cell death by apoptosis.^[^ ^161^ ^]^ So, Rexin‐G encodes a CCNG1 inhibitor gene to be able to kill rapidly dividing cells, cancer cells, and displays a SIG (Signature)‐decapeptide to bind to anaplastic SIG proteins which are expressed in the tumor environment.^[^ ^162^ ^]^ Technically, Rexin‐G is “disease‐seeking” or pathotropic and is a murine leukemia virus (MLV)‐based nanoparticle. It encodes an N‐terminal deletion mutant construct of human cyclin G1 gene that is expressed under a hybrid LTR promoter.^[^ ^163^ ^]^ ReltaVax (DeltaRex), also encodes two important genes: a GM‐CSF gene for in situ autoactivation, and the HSVtk gene to regulate GM‐CSF expression in cancer cells.^[^ ^164^ ^]^ This treatment was tested in phase 1 and phase 2 studies including over 280 cancer patients around the world, and it has proven efficacy in patients with advanced cancers such as osteosarcoma, breast cancer, malignant peripheral nerve sheath tumor, pancreatic cancer, and B‐cell lymphoma.^[^ ^165^ ^]^

Gene therapy can be described as a tool to return back and correct tumor‐induced gene mutations into their native wild forms.^[^ ^166^ ^]^

### 3.4 CAR‐T Therapy

Nowadays, immunotherapy is known to be a potent antitumor weapon.^[^ ^167^ ^]^ A revolution in immunotherapy has launched an innovation known as the Chimeric antigen receptor modified T (CAR‐T) cells.^[^ ^168^ ^]^ This new technology has proven to be the most promptly developed and extensively applicated arm of anticancer cellular immunotherapy.^[^ ^169^ ^]^ More specifically, CAR‐T therapy has succeeded remarkably in the treatment of hematological malignancies, mainly acute lymphoblastic leukemia (ALL), and large B‐cell lymphomas.^[^ ^170^ ^]^ In August and October 2017, two CD19‐targeting CAR‐T cell techniques Kymriah and Yescarta were approved by the FDA to treat B‐ALL and diffuse large B‐cell lymphoma (DLBCL).^[^ ^171^ ^]^

CARs are synthetic receptors consisting of four main elements:1) an extracellular target antigen‐binding domain, 2) a hinge region, 3) a transmembrane domain, 4) one or more intracellular signaling domains.^[^ ^172^ ^]^ The main component of the extracellular antigen recognition domain is the single‐chain variable fragment (scFv) of the target antigen‐antibody. The scFv consists of the heavy chain variable regions (VH) and the light chain variable regions (VL) of the specific antibody to the tumor‐associated antigen (TAA).^[^ ^173^ ^]^ CAR‐T cells are genetically manufactured to take out the patient's T‐cells, and expressing single‐chain antibodies that exclusively recognize and bind to cancer cells antigens, such as CD‐19.^[^ ^174^ ^]^ Practically, blood is collected from the patient or an allogeneic donor, it undergoes apheresis, purification, and then genetic engineering. T‐cells are then expanded ex vivo and tranfused back to the patient.^[^ ^175^ ^]^ In brief, CAR is a recombinant receptor construct enabling the redirection of the T‐cell‐mediated cytotoxicity to cancer cells in an HLA‐independent manner.^[^ ^176^ ^]^ In order to eliminate the expression of HLA classes 1 and 2 on allogeneic T cells, genetic engineering is done with viral or non‐viral techniques.^[^ ^177^ ^]^ In a more simplified manner, a patient's Tcells are modified in a way to express an engineered receptor capable of recognizing a specific antigen on cancer cells.^[^ ^178^ ^]^

Nonetheless, the CAR‐T therapy is not without any risks, severe toxicities can emerge after the infusion of the cells into the patients, such as cytokine releases syndrome (CRS), neurotoxicities, graft‐versus‐host disease (GVHD), tumor lysis syndrome, and on‐target/off‐tumor toxicity.^[^ ^179^ ^]^ Thus, further progress in the CAR‐T technology is of crucial necessity.^[^ ^180^ ^]^

#### 3.4.1 CAR‐T Therapy – Gene Silencing

The CRISPR technology is an RNA‐guided DNA‐cleavage technique that was first discovered in *E. coli* bacteria as an adaptive immune response against foreign DNA. The most widely used CRISPR type is the type II CRISPR/Cas9 system. The latter targets DNA sequences by a single Cas protein from the Streptococcus pyogenes bacteria (SpCas9).^[^ ^181^ ^]^ Among the Transcription activator‐like effector nucleases (TALENs), CRISPR/Cashas proven to be the most competent gene editing tool.^[^ ^182^ ^]^ In the context of CAR‐T cells therapy, the CRISPR technology has been a promising tool to improve the drawbacks of CAR‐T cells therapy.^[^ ^183^ ^]^ For instance, CRISPR/Cas9 can be used to disrupt immune checkpoints like the PD‐1 gene (PDCD‐1), or the Cytotoxic T‐lymphocyte associated Antigen‐4 (CTLA‐4). In fact, the disruption of PD‐1 increases the cell lysis capacity. Using this methodology, it was shown that mice cancerous cells were eliminated for up to 28 days. The TGF‐β receptor II can be modified in the same manner as PD‐1 enabling the multiplication of CAR‐T cells in mice and thus inhibit tumor development.^[^ ^184^ ^]^ On another note, and to lower the above‐mentioned toxicity of CAR‐T cells, CRISPR can remove genes that encode cytokines responsible for neurotoxicity and cytokine release syndrome (CRS) like GM‐CSF and IL‐6. The knockout of the IL‐6 gene was successful in ameliorating CRS‐like toxicity in leukemia‐bearing mice.^[^ ^185^ ^]^ Nevertheless, in CD33 or CD123 induced CAR‐T cell therapy preclinical studies, it was reported that myeloid‐directed immunotherapy eradicates normal and malignant cells causing bone marrow failure, making CAR‐T cell therapy a barrier in treating acute myeloid leukemia (ALL). To tackle this issue, new approaches have been implemented using a potent anti‐CD33 CAR‐T cells followed by infusions of CRISPR/Cas9‐modified CD33‐knockout normal hematopoietic stem cells (HSCs). This will allow an antigen‐specific cytotoxicity with reconstruction of efficient hematopoiesis.^[^ ^186^ ^]^

#### 3.4.2 CAR‐T Therapy – Suicide Gene Therapy

Another method used to combat CAR‐T cells therapy related toxicities, most importantly CRS and CRES, is the Suicide Gene Therapy. Suicide genes such as inducible Caspase 9 (iCasp9, iC9), human thymidylate kinase (TMPK), and herpes simplex virus tyrosine kinase (HSV‐TK), are used to deplete CAR‐T cells.^[^ ^187^ ^]^ Those genes are elements that are genetically encoded, are incorporated in the T‐cells, and are activated by the administration of a pharmaceutical agents.^[^ ^188^ ^]^

For instance, the iC9 suicide gene includes the intracellular part of the human caspase 9 protein, fused to a drug‐binding domain derived from the human FK506‐binding protein.^[^ ^177^ ^]^ Rimiducid, an inert biomolecule AP1903, is given intravenously to incite the downstream apoptosome.^[^ ^189^ ^]^ Elimination is realized by the activation of the caspase 3 apoptotic pathway in cells expressing iC9 and TMPK. Moreover, in patients receiving haploidentical stem‐cell transplants (HSCT), the iC9 was successful. T‐cells expressing iC9 were immediately depleted at onset of GVHD.^[^ ^190^ ^]^ Indeed, it has been shown that the use of iC9 in CAR‐T therapy eliminates those cells both in vivo and in vitro, making this strategy a powerful rescue in case of inadvertent blast‐cell transduction during CAR‐T cell manufacturing.^[^ ^191^ ^]^ Concerning the HSV‐TK, ganciclovir (GCV) was found to be the most potent pro‐drug (as pharmaceutical agent used for activation). After taking GCV, HSV‐TK will catalyze the phosphorylation of GCV producing a toxic GCV‐triphosphate leading to a competitive inhibition of guanosine incorporation and thus inhibiting DNA synthesis and ending up with cellular death.^[^ ^192^ ^]^

#### 3.4.3 CAR‐T Therapy – Anti‐Tumor Angiogenesis

CAR‐T cell therapy has proven its efficacy mainly against hematological malignancies. However, this novel technology did not its potency in solid tumors yet. This is due to either the incapacity of the administered cells to migrate and reach the inside of the solid tumors, those cells can only reach the surface of the tumor and some limited areas, or due to the formation of an immunosuppressive environment by the tumor cells protecting them against therapy.^[^ ^193^ ^]^ One of these physical environments is the vascular barrier which forms a vascular matrix around the tumor making the access more difficult.^[^ ^194^ ^]^ Like any other living cell, tumor cells rely on angiogenesis to prosper and keep on multiplying.^[^ ^195^ ^]^ For a decent response, CAR‐T cells must:1) infiltrate into and homogeneously spread all through the tumor microenvironment (TME) in enough quantities, 2) enact powerful and localized anti‐tumor function and, 3) persist and proliferate in TME.^[^ ^196^ ^]^

To test the efficacy of vascular‐targeted therapy in serving CAR‐T therapy some steps must be considered. First, drugs should be tested for their capacity to disrupt a vascular microenvironment in solid tumors. Vascular disrupting agents (VDAs) are antiangiogenic drugs capable of stimulating apoptosis in tumor cell‐associated vascular endothelial cells. This is done by disturbing the microtubule polymerization stability of cells causing damages to the vascular system and thus cutting down the blood supply of tumor cells and leading to necrosis.^[^ ^197^ ^]^ Second, VDAs should be tested for their ability to promote the infiltration of CAR‐T cells into the solid tumor and boost the desired therapeutic effect. A classical VDA that is able to inhibit microtubule polymerization is Combretastatin A‐4 phosphate (CA4P).^[^ ^198^ ^]^ The latter selectively blocks the signaling pathway of endothelial cell‐specific connexin VE‐cadherin as it has a high selectivity for tumor vasculature.^[^ ^199^ ^]^ Third, CA4P should be tested for its efficacy in developing a good therapeutic effect of the CAR‐T cells on solid tumors. Indeed, it was shown that CA4P improves the antitumor work of CAR‐T cells in solid tumors promoting their infiltration in many tumors in vivo. Merging CA4P and CAR‐T is a promising approach and technique for the treatment of solid tumors (**Table** **2**).^[^ ^200^ ^]^

**Table 2:** Summary of major limitation of CAR T‐cell therapy and potential strategies to overcome limitations.^[^ ^172^ ^]^

Limitations of CAR‐T cell therapy	Potential strategies
Antigen escape	Targeting multiple antigens (dual or tandem CARs)
	Preliminary dinical trial resuhs of CD19/CD22 targeted CARs for treatment of ALL/DLBCL and CD19/BCMA targeted for multiple myeloma have demonstrated promising efficacy*'.
	Solid tumer: HER2/IL13Ra2 (glioblastoma) and HER2/MUC] (breast cancer) CARS produce superior antitumor responses Compared to single target therapy?
On‐target off‐tumor effects	Targeting tumor‐restricted post‐transiational modifications
	‐Four major CAR‐T cell targets have been investigated TAG72TM, B7‐H3, MUCI', and MUCI6TM
(CAR‐T cell trafficking and tumer infiltration	Local administration vs systemic delivery
	Superior therapeutic efficacy of intrapleuralTM and intraventriculas*’TM injection of CAR‐T cells in mesothelioma and glioblastoma/brain cancer patients, respectively.
	Expressing chemokine receptors on CAR‐T cells that match and respond to tumor‐derived chemokines sintegrin avQ6‐CAR‐T ceils modified to express CXCR2 of CAR‐T cells overexpressing CXCRI/CKCR2
	Engineering CAR‐T ceils to enhance penetration through physical barriers (tumor stroma) <CAR‐T cells that express heparanase or fibroblastactivation protein targeted CAR‐T cells have shown enhanced infitration and antitumor activity.
Immnunosuppressive microenvironment	Combination immunetherapy with CAR‐T ceils and checkpoint blockade hematolagical malignancy, combination PD‐1 blockade and CD19 CAR‐T cell therapy in B‐ALL patients improved outcomes and improved CART cell persistence.
	Engineering CAR‐T ceils to provide immunestimulatory signals in the form of cytokines or CARs resistant to immunosuppressive factors
	Engineering CARs to provide immunostimulatory signals have relied on IL‐12 secretion, IL‐15 expression, and redirecting immunosuppressive cytokines (eg. IL‐4) resulting in increased survival CARS resistant to immunosuppressive factors in the hostile tumor microenvironment such as TGF f‐mediated inhibitory signals have been developedTM.
CAR‐T celbassodated toxicities	Altering CAR structure to ameliorate toxicity Decreasing CAR antigen‐binding domain affinity to micromolar affinity.
	Cytokine secretion can be modulated by modifying the CAR hinge and transmembrane regions.
	Tailoring the costimulatory domain based on tumor type, tumor burden, antigen density, target antigen‐antigen binding domain pair, and concems of toxicityTM.
	CAR immenunogenicity can be decreased by utilizing human/humanized antibody fragments instead of murine‐derived CARS*°TM.

## 4 Therapeutic Challenges Perspective and Conclusion

The development, diversification, and investments in the gene therapy point to a bright and optimistic future for these treatments for human illnesses in the following years. However, there are still a lot of technical issues that need to be addressed. Major challenges in gene therapy currently include low efficacy and safety concerns, immune system reactions, unclear gene manipulations and therapy guidelines, sophisticated approaches for drug development and manufacturing, mysterious host‐drug interactions, and high costs.

For the creation of safe gene‐therapy products suitable for gene modification, it is mandatory to control both in‐vivo and ex‐vivo therapeutic procedures. To overcome innate immunity and improve the effectiveness of gene therapy, novel viral vectors, tailored viral strains, and improved non‐viral gene delivery techniques should be developed. The therapeutic endurance of gene treatments would also be increased by enhancing their systemic half‐life. On the other hand, gene editing should be governed by international law and well regulated.

Genome editing will change the future of cancer treatment, from generalized cancer treatment strategies toward a more personalized approach in cancer treatment, based on the patient's genome, immune status, and the genetic profile of the tumor.

## Conflict of Interest

The authors declare no conflict of interest.

## Author Contributions

J.G., A.C., H.K., C.H., A.I., K.M., M.M., F.K., D.G.H., P.N., and C.K. wrote and approved the manuscript.
